# Responses of *Salmonella* biofilms to oxidizing biocides: Evidence of spatial clustering

**DOI:** 10.1111/1462-2920.16263

**Published:** 2022-11-06

**Authors:** Kerry Guest, Thomas Whalley, Jean‐Yves Maillard, Andreas Artemiou, Barbara Szomolay, Mark A. Webber

**Affiliations:** ^1^ School of Pharmacy and Pharmaceutical Sciences Cardiff University Cardiff UK; ^2^ School of Biosciences Cardiff University Cardiff UK; ^3^ School of Mathematics Cardiff University Cardiff UK; ^4^ School of Medicine Cardiff University Cardiff UK; ^5^ Quadram Institute Bioscience Norwich Research Park UK; ^6^ Norwich Medical School University of East Anglia Norwich Research Park UK

## Abstract

The spatial organization of biofilm bacterial communities can be influenced by several factors, including growth conditions and challenge with antimicrobials. Differential survival of clusters of cells within biofilms has been observed. In this work, we present a variety of methods to identify, quantify and statistically analyse clusters of live cells from images of two *Salmonella* strains with differential biofilm forming capacity exposed to three oxidizing biocides. With a support vector machine approach, we showed spatial separation between the two strains, and, using statistical testing and high‐performance computing (HPC), we determined conditions which possess an inherent cluster structure. Our results indicate that there is a relationship between biocide potency and inherent biofilm formation capacity with the tendency to select for spatial clusters of survivors. There was no relationship between positions of clusters of live or dead cells within stressed biofilms. This work identifies an approach to robustly quantify clusters of physiologically distinct cells within biofilms and suggests work to understand how clusters form and survive is needed.

**Significance statement:**

Control of biofilm growth remains a major challenge and there is considerable uncertainty about how bacteria respond to disinfection within a biofilm and how clustering of cells impacts survival. We have developed a methodological approach to identify and statistically analyse clusters of surviving cells in biofilms after biocide challenge. This approach can be used to understand bacterial behaviour within biofilms under stress and is widely applicable.

## INTRODUCTION

Biocides are crucial for control of microbial contamination and infection and are used in a wide range of clinical, industrial, veterinary, and domestic settings (Linley et al., 2012). Whilst many common biocidal agents have been employed for decades there are still major gaps in our understanding of mechanisms of action and resistance.

Oxidizing biocides have a broad spectrum of activity, similar chemistries, and proposed mechanisms of action. The basic mechanism by which they exert their biocidal activity is thought to be via damage to cellular macromolecules (Finnegan et al., 2010). They are usually low molecular weight compounds able to enter the bacterial cell, thereby accessing intracellular targets, although there is also evidence for antimicrobial action exerted at the cell wall and membrane for some compounds (Finnegan et al., 2010). The three most used common oxidizing biocides are hydrogen peroxide, peracetic acid and sodium hypochlorite.

Hydrogen peroxide (H_2_O_2_) is widely used for disinfection, sterilization and antisepsis and degrades into non‐toxic by‐products of water and oxygen making it an attractive choice for any application involving food production (Rutala & Weber, 1999). The biocidal activity of hydrogen peroxide may be due to interactions with intracellular iron forming iron ions and hydroxyl radicals (Finnegan et al., 2010) formed by the Fenton reaction. Several studies have shown hydrogen peroxide to be responsible for damage to DNA (Henle & Linn, 1997) proteins (Imlay et al., 1998), amino acids (Dean et al., 1997), and cell membranes (Baatout et al., 2006; Brandi et al., 1991; Peterson et al., 1995).

Peracetic acid (PAA) is a peroxide of acetic acid (Block, 1991). It is soluble in water and exists in equilibrium between acetic acid (CH_3_CO_2_H), peracetic acid (CH_3_CO_3_H), water (H_2_O) and hydrogen peroxide (H_2_O_2_). It is a weak acid with a p*K*_a_ of 8.2 (Unis, 2010). It has greater oxidizing potential than chlorine or chlorine dioxide and is environmentally safe due to its degradation into non‐toxic components (Kitis, 2003). It also has higher antimicrobial activity than hydrogen peroxide (Wagner et al., 2002) and remains active in the presence of interfering matter including organic material such as blood (Russell & McDonnell, 1999). The mechanism of action of peracetic acid has not been fully investigated but it has been hypothesised that it disrupts sulphydryl (—SH) and sulphur (S—S) bonds in biomolecules (Russell & McDonnell, 1999). It has also been suggested that PAA disrupts the cell wall and cell membrane by oxidizing structural lipoproteins and when acting intracellularly may inactivate vital metabolic enzymes and DNA bases (Kitis, 2003).

Chlorine was first discovered by Scheele in 1774 (Rutala & Weber, 1997) and has a wide range of antimicrobial activity. Sodium hypochlorite (NaClO) is a salt of the hypochlorite ion dissolved in water which in solution can dissociate to give hypochlorous acid. Hypochlorous acid has considerably more antimicrobial activity than the hypochlorite ion and is responsible for much of the antibacterial efficacy (Rossi‐Fedele et al., 2011). Sodium hypochlorite has a broad spectrum of activity with its primary industrial use being water treatment (Rutala & Weber, 1997). Proteins, peptides, lipids and DNA have all been shown to be oxidized by sodium hypochlorite at physiological pH with C=C double bonds, peptide bonds, peptide groups and thiol groups susceptible to electrophilic damage (Fukuzaki, 2006). Notwithstanding this reactivity, it is postulated that the primary action of the biocide is oxidative damage to DNA synthesis since low concentrations of sodium hypochlorite leave protein synthesis far less affected than DNA synthesis (Russell & McDonnell, 1999).

Concerns have been raised about the possibility of biocide resistance emerging. It is generally not possible for bacteria to achieve resistance to in use biocide concentrations, but studies have documented strains able to survive low levels of biocide (tolerance) and identified mutants isolated after biocide exposure with cross resistance to antibiotics (Copitch et al., 2010; Karatzas et al., 2008; Randall et al., 2007; Walsh et al., 2003). Bacterial biofilms are far more resistant to biocide challenge than planktonic cells (Bansal et al., 2019; Russo et al., 2013; Vestby et al., 2009). The reasons for these significant changes in bacterial susceptibility to biocides have been proposed to relate to the structure of the bacterial biofilm and the EPS matrix, altered metabolism in the biofilm phenotype, persister cell generation and an increase in genetic transfer.

Biofilms as a community of cells encounter a range of internal and external stresses resulting from nutrient limitation, waste product secretion, ecological competition, desiccation, and antimicrobial exposure (Hall‐Stoodley et al., 2004). Biofilms are inherently resilient to stress (Rode et al., 2020) in part due to the heterogeneity of cells and structures within the community, cells can be spatially organized into clusters and this has been linked to antimicrobial survival (Wong et al., 2021). To characterize the spatial distribution of cells, digital image analysis (Dazzo & Yanni, 2017; Schillinger et al., 2012), spatial analysis methods such as Ripley's K (Hart et al., 2019; Ishkov et al., 2021; Marchal et al., 2017) and methods for determining spatial randomness such as the Hopkins statistic (Drury et al., 1993; Espinoza et al., 2012) have been used. However, methods for evaluating clusterability can vary significantly and a comprehensive comparison of statistical tests in the biofilm context is currently lacking.

The aim of this study was to employ a combination of microscopy and statistical approaches to identify cell survival and the impact of spatial distribution of cells within a biofilm on biocide susceptibility. We investigate the effect of three oxidizing biocides against two *Salmonella enterica* subsp. enterica strains (one serovar Typhimurium and one Agona) with different biofilm forming capacities. First, with a support vector machine approach, we showed spatial separation between the two strains. Second, we applied five statistical tests, four of which are new in the biofilm setting, and used HPC, to evaluate clusterability. Our results indicate that biocides of medium potency, like NaClO, have a stronger tendency to select for spatial clusters of surviving cells in *S*. Typhimurium biofilms. Finally, we showed that simple visualizations of confocal images can efficiently quantify colocalization between live and dead cells and that there is no link between the spatial locations of live and dead clusters of cells within stressed biofilms.

## EXPERIMENTAL PROCEDURES

### Bacteria and growth conditions

Two strains of *Salmonella* were used in this study, *Salmonella* Typhimurium SL1344, a commonly used reference strain and *Salmonella* Agona 3750, a persistent isolate associated with food spoilage kindly provided by Unilever, UK Strains of *S*. Agona are commonly reported as strong biofilm formers, associated with persistence in food production environments (Aaron et al., 2018; Diez‐Garcia, 2012) and 3750 demonstrated a high capacity for biofilm formation (Figure S1). Both strains were routinely cultured using either tryptone soya agar (Oxoid, Basingstoke, UK) or tryptone soya broth (Oxoid, Basingstoke, UK) and incubated aerobically at 37°C (±1°C), for 24 hours in an incubator (Memmert INE 600, Schwabach, Germany). For biocide challenge, a MOPS (3‐(*N*‐morpholino) propanesulfonic acid) based minimal medium (VWR, Lutterworth, UK) was used to simulate the low nutrient environment often associated with formation of biofilms and to limit the presence of organics that the oxidizing biocides could interact with for susceptibility testing. Growth media was supplemented with 400 mg/L histidine (Sigma, Dorset, UK) as *Salmonella* Typhimurium SL1344 is a histidine auxotroph.

### Biocides

Biofilms of both strains were grown for 24 and 48 h on stainless steel disks in MOPS +400 mg/L histidine at 37°C. Biofilms were either untreated (control) or exposed to three oxidizing biocides for 20, 40, 60 min: hydrogen peroxide (H_2_O_2_; 25,000 ppm), peracetic acid (PAA; 30 ppm) or sodium hypochlorite (NaClO; 40 ppm).

All three biocides were bought in solution (Sigma, Dorset, UK) and the concentration of sodium hypochlorite and hydrogen peroxide were assayed weekly. Peracetic acid was not assayed as it was guaranteed by the manufacturer to remain at the supplied concentration for 6 months after opening. Neutralisers were used to quench the activity of the biocide, when investigating the bactericidal activity of the agent over time. The neutralisers used and their components were 250 ppm catalase for hydrogen peroxide, universal neutraliser for peracetic acid and 10,000 ppm sodium thiosulphate for sodium hypochlorite.

### Biofilm viability and microscopy

Viability of cells within biofilms after biocide exposure was determined using a Bioflux microfluidic system (Fluxion, USA). Bacterial cell suspensions were grown overnight and diluted to an OD of 0.1 at 600 nm in MOPS based minimal media +400 mg/L histidine. Fifty microlitres of this was then used to inoculate flow cells and bacteria were left in static conditions for 1 h to attach at room temperature (19 ± 1°C). For the duration of experiments media was supplied to the flow cells at a flow rate of 0.3 dyne at room temperature (19 ± 1°C). After biofilms were established, at each test point biocides were introduced into desired flow cells at a rate of 0.3 dyne for either 20, 40 or 60 min. All experiments were replicated in three independent flow cells.

**FIGURE 1:**
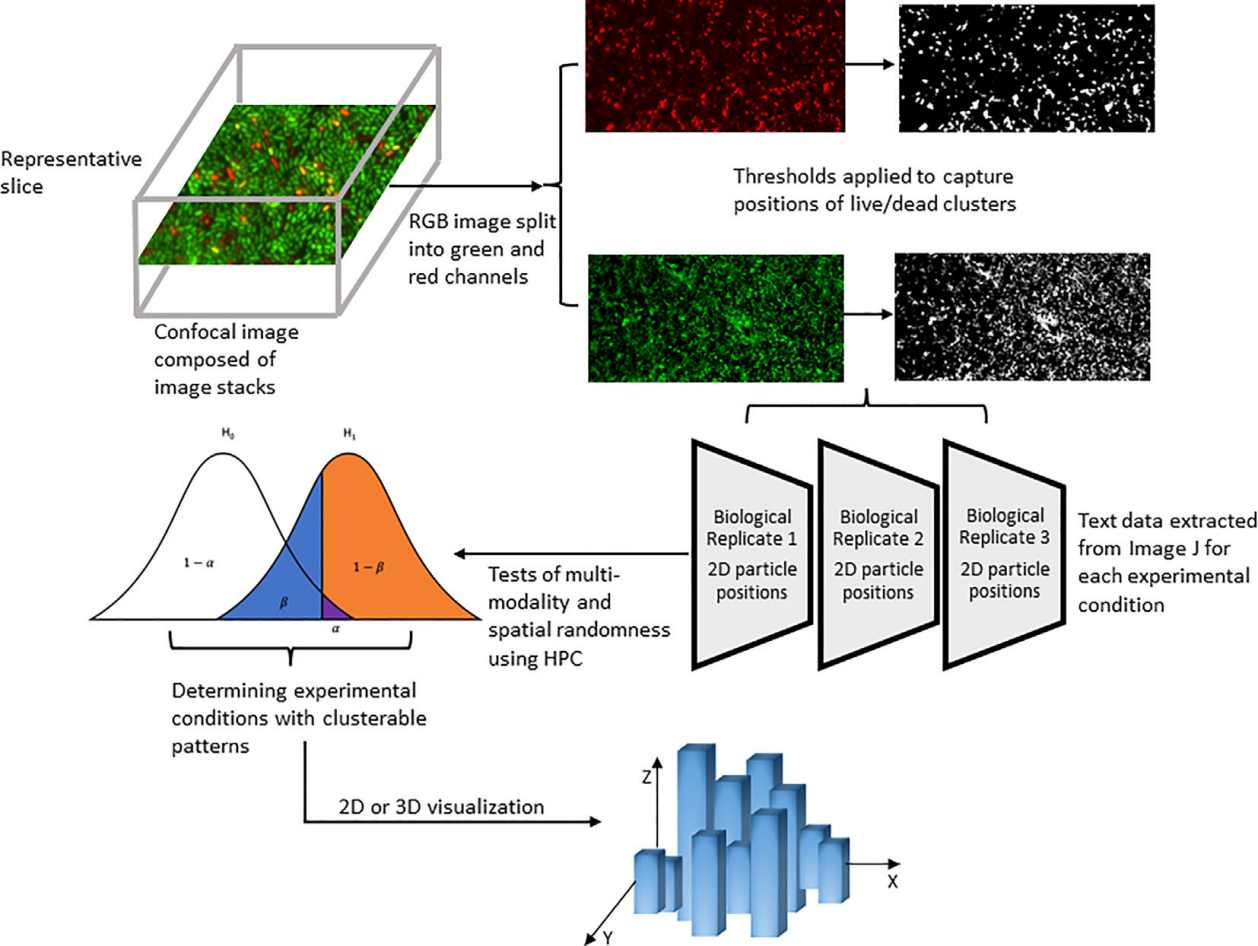
Overview of the data analysis workflow

Biofilms were visualized with both phase contrast and fluorescent microscopy. Images were taken before biocide exposure, after biocide exposure and after 24 h of growth post‐biocide exposure. For live/dead staining, two fluorescent dyes were added to the outlet well: 748 μM PI (Sigma, Dorset, UK) and 10 μM SYTO 9 (Life Technologies, Leicestershire, UK) diluted in 1 ml in PBS and flowed to the input well at 2 dyne for 5 min. Fluorescence images were captured using a Zeiss LSM 710 Confocor 3 inverted confocal microscope (Carl Zeiss ltd., Germany) viewed at 63× under oil. The 488 nm laser was used at 30% power and using the MBS 488/543/633 filter. The range used for SYTO 9 was 500–550 and for PI was 600–650 (Germany) at 63×. Images were taken in triplicate from each flow cell across different fields of view (the left, middle and right). For Z‐slices, images were taken at intervals of 0.5 μm across the depth of the biofilm. Biofilms were imaged after treatment with biocides and compared to replicate, untreated control biofilms (Figure 1).

Light microscopy images were analysed using the Bioflux EZ software (Labtech, Sussex, UK) percentage coverage tool and highlighting three different fields of view to give an average value for percentage coverage for quantitative comparison. Fluorescent RGB tiff images were analysed using ImageJ (National Institutes of Health, Maryland, USA). Images from the bottom slice of each stack were chosen to avoid and selection bias and were split into colour channels and the green and red channels thresholded using the automated ImageJ thresholding algorithm to identify cells from background. Particle properties were extracted using a cut‐off of two pixels to identify cells (which are bigger than this but this was a minimum size to remove background noise) and particles bigger than this were recorded to .csv files (particle number, area, mean signal fluorescence intensity), min and max signal, middle *X* and *Y* coordinates and particle perimeter length (in pixels) for spatial analysis. *Z*‐stacks were captured, and slices taken at intervals of 0.5 μm across the depth of the biofilm. The csv files of the live cells for 19 different conditions were used for statistical analysis (Table S1).

## RESULTS AND DISCUSSION

### Weak and strong biofilm forming strains can be distinguished by support vector machine‐based differentiation using confocal microscopy images

**FIGURE 2:**
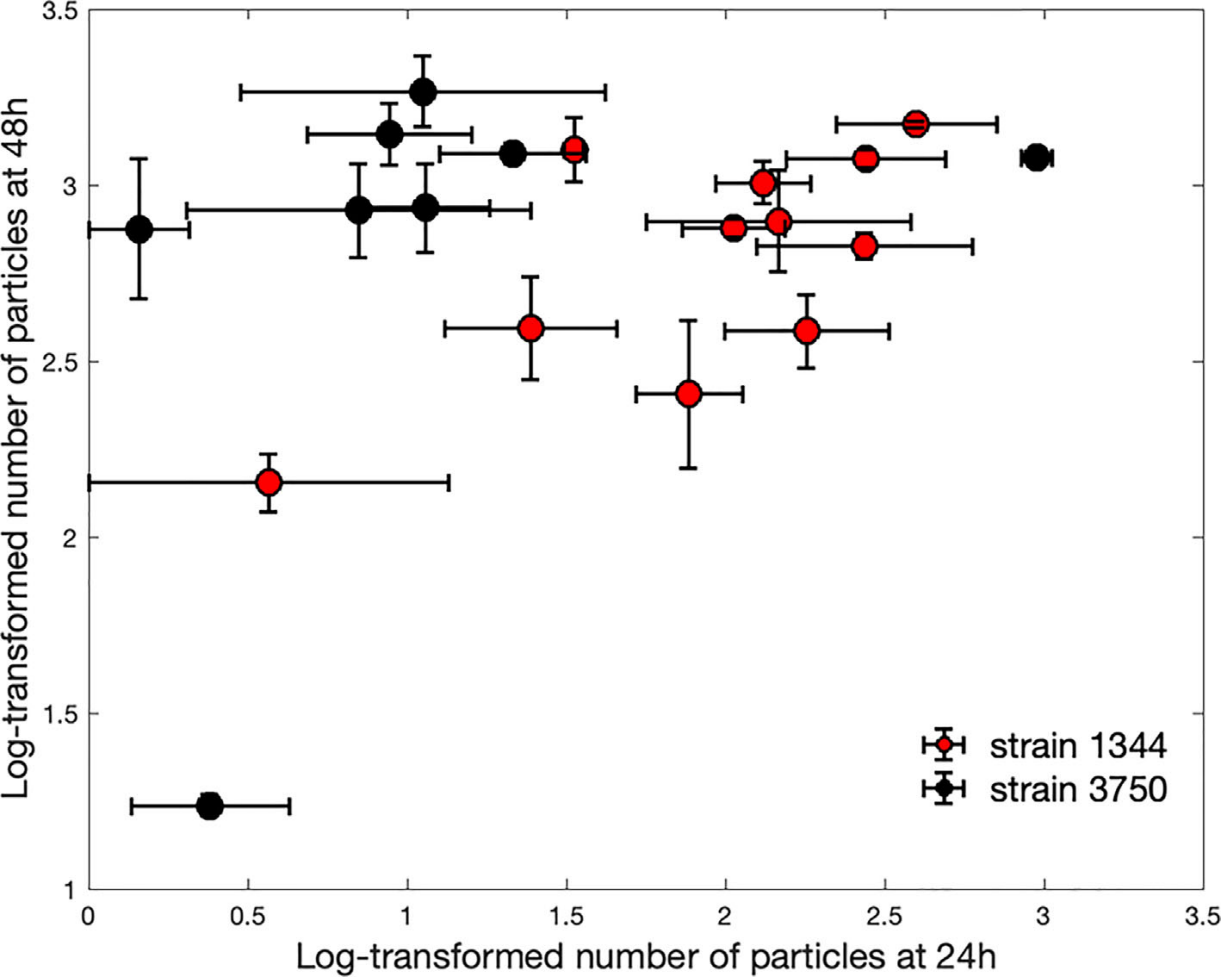
Mean and SE of the number of particles for 19 different conditions (Table S1). Red and black colours show discrimination between weak (SL1344) and strong (3750) biofilm forming strains, respectively. To assess whether the underlying 1D probability distributions between the two strains differ, we performed two‐dimensional two‐sample Kolmogorov–Smirnov (KS) test, a generalization of the classical KS test, using the transformed data with coordinates denoting the mean number of particles at 24 h and 48 h (Fasano & Franceschini, 1987). The difference between the mean number of particles in the 2D space (24 h, 48 h) from strains SL1344 and 3750 was statistically significant. *p*‐value < 0.01).

We used confocal microscopy images to identify live and dead cells (based on staining with SYTO‐9 and propidium iodide) for two *Salmonella* strains with different biofilm forming capacities (*S*. Typhimurium SL1344, a relatively modest biofilm former, and *S*. Agona 3750, a strong biofilm forming strain, Figure S1). We then aimed to compare the ability of each to survive exposure to oxidative biocides. Our first goal was to quantify differences in biofilm formation from image data. For each image, the number of particles (cells) were extracted, added unity and log10‐transformed. Mean and standard error (SE) of flow cell coverage for both strains at 24 and 48 h are shown in Figure 2 and confirmed the expected greater increase in biomass in strain 3750 versus SL1344 (representative images and average flow cell coverage are shown in Figure S1).

Support vector machines (SVM) are learning algorithms which aim to identify a function (hyperplane) that can separate datasets. Biomedical applications of SVM include the classification of bacterial species to distinguish between disease conditions (Yoram et al., 2018) or the innate fluorescence signatures of microbial cells (Yawata et al., 2019), prediction of biofilm‐inhibiting‐peptides (Gupta et al., 2016), classification of antibiotics (Jung et al., 2014) or differentiation of human and in vitro biofilm transcriptomes (Cornforth et al., 2018). Here we applied an SVM classifier with radial basis function (RBF) kernel to differentiate between the weak (SL1344) and strong (3750) biofilm forming strains using the mean number of particles from a single 2D plane of Z‐stack for each condition.

The data were split into 58% training (11 data points) and 42% test (8 data points). We estimated γ, the RBF kernel parameter, directly from data points in the training set **X** $\subset R^{11\times 2}$, using the formula in Li et al. (2011)

γ=1τ2,τ=1112∑i<j,j=211Xi−Xj2,

where ||·|| is the Euclidean distance and *τ* ^2^ is an estimate of the variance in the data and $X_{i}$ is the *i*th observation used in the sample, consisting of the mean number of particles at 24 and 48 h in a given Z‐stack.

Then, for a fixed γ= 0.27, 10‐fold cross‐validation was performed to select the best misclassification cost, *C*, using the tune function from the e1071 library (https://cran.r-project.org/web/packages/e1071/index.html). For the optimal *C* = 12%, 35% of test observations are subject to misclassification. The level of prediction accuracy on the test data is shown by calculating the receiver operating characteristics (ROC) curve in Figure S2.

**FIGURE 3:**
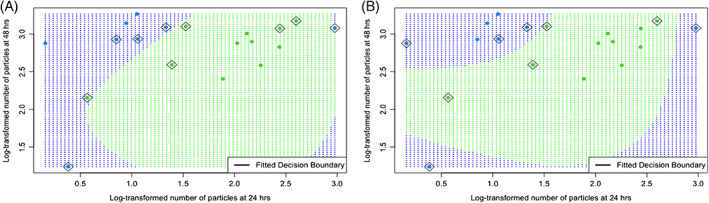
Decision boundary generated by the SVM classifier and support vectors in diamonds. The mean number of particles could distinguish between weak and strong biofilm forming strains with relatively high accuracy. *γ* = 0.27, *C* = 12 in a, and *γ* = 0.8, *C* = 5 in B.

The SVM algorithm tries to create a decision boundary such that the separation between two classes of data is as wide as possible. The misclassified points and those closest to this hyperplane are the support vectors. These are shown in Figure 3A as well as the fitted decision boundary that was generated by the RBF kernel by learning from data. The data show the SVM has generated a clear separation between the green and blue data points corresponding to the SL1344 and 3750 biofilms respectively, further confirming their inherent difference in biofilm formation.

We also performed 10‐fold cross‐validation for both parameters γ and *C*. For γ = 0.8, *C* = 5, the decision boundary correctly classified all training points. Although only 15% of test observations are subject to misclassification and Figure 3B is seemingly more accurate than Figure 3A, these results are less robust. This is because several runs of 10‐fold cross‐validation are required for correct binary classification.

To evaluate the performance of classification algorithms for Figure 3A, we used leave‐out‐one cross‐validation (LOO‐CV), when a partition is made up of 18 training data and 1 testing. This is a special case of *K*‐fold validation, when *K* equals the number of data points in the set. The parameters γ and cost of constraints violation were estimated using the same approach as above 19 separate times. Except 2, all data points were correctly classified by LOO‐CV, thus predicting a high (89.5%) classification accuracy. Our results show that the SVM approach could accurately distinguish between strains with different biofilm forming capacities using confocal image data at 24 and 48 h, regardless of treatment.

### Microbial colonies within *Salmonella* biofilms are non‐randomly distributed

Non‐random spatial organization of cells and matrix components are believed to contribute to the persistence of biofilm communities as these structures provide structurally distinct microenvironments as well as promoting physiological heterogeneity of cells within a community (Petersen et al., 2019). In support of this, a non‐uniform distribution of surviving cells within *Salmonella* biofilms after biocide challenge was observed by confocal microscopy (Figure S3) and this differed between test biocides. We used both spatial randomness tests (Hopkins statistic), multimodality tests (Classic Dip test and Classic Silverman test) and related tests on reduced versions of the data (PCA Dip and Dip‐dist) to study and evaluate the clusterability of survivor positions in the different conditions. These tests were applied to the 2D plane of Z‐stack.

Hopkins statistic tests for spatial randomness and evaluates if any feature is distributed non‐randomly across the data set (Hopkins & Skellam, 1954; Lawson, 1990). Although Hopkins may be preferred when small clusters are of interest (Adolfsson et al., 2017), it has only been used in a few biofilm‐ or receptor aggregate‐related studies. Examples are studies on the interaction of latex particles with *P. aeruginosa* (Drury et al., 1993) and the quantification of clustering of membrane proteins labelled with gold nanoparticles (Espinoza et al., 2012). The ‘hopkins’ function from the clusterend R package (Adolfsson et al., 2017) was used to calculate the Hopkins statistic for each of our conditions, which distinguishes non‐clustered from moderately or highly clustered distributions.

The Classic Dip test rejects the null hypothesis of unimodality if the empirical distribution is sufficiently different from the closest possible uniform one (Hartigan & Hartigan, 1985). The Classic Silverman test is based on the kernel density estimate and it will reject the assumption of unimodality if a mixture of distinct Gaussian distributions is required to produce the underlying empirical distribution (Silverman, 1981). The ‘modetest’ function from the multimode R package (https://cran.r-project.org/web/packages/multimode/multimode.pdf) and the ‘dip.test’ from the diptest R package (https://cran.r-project.org/web/packages/diptest/diptest.pdf) were used for Silverman's mode estimation method and Hartigan's dip statistic.

PCA Dip (Dip test on principal components) and Dip‐dist (Dip test on pairwise distances) uses the classic Dip test to test whether the first principal component is multimodal (Adolfsson et al., 2017) or to test for clusters on a set of pairwise distances (Kalogeratos & Likas, 2012). The codes for PCA Dip and Dip‐dist were implemented using the diptest R package by using the first principal component and the matrix of pairwise distances.

We ran all five methods with default parameters in a series of simulations to evaluate the clusterability of data using HPC. For the multimodality tests 1000 runs for the Classic Dip test, PCA Dip, Dip‐dist and 100 runs for the Classic Silverman test were performed. The percentage of data sets on which the tests yielded a *p*‐value less than 0.05 was recorded, indicating that the test rejected the assumption of unimodality at 5% significance level. For unambiguously unclusterable image datasets, the proportion of rejections corresponds to a Type I error (the rate of incorrectly classifying unclusterable data sets as clusterable) (Adolfsson et al., 2017).

For the Hopkins statistic, clusterability can be inferred from a threshold based on the Beta distribution. Under a null hypothesis, the test statistic H will follow a Beta distribution with both parameters equal to n, the number of data points sampled (Adolfsson et al., 2017; Hopkins & Skellam, 1954; Lawson, 1990). Hence, the Beta statistic should be compared to the Beta quantile *qα*(*n*,*n*) *α*, which is defined as the probability of concluding that the data is clustered, assuming it was generated without clusters, that is, *p*(*H* < *qα*(*n*,*n*)) is 100*α*%. The recommended sampling rate for *n* is 5%–10% of the data (Lawson, 1990). We randomly sampled 10% of the data in a series of 1000 runs and following (Adolfsson et al., 2017), we used a one‐sided test with *α* = 0.05.

Each of these methods can capture the structure of the data differently, to take a consistent approach, we considered data from a given experimental condition clusterable if a replicate has at least 25 particles and the clusterability fraction exceeds 80% in at least two biological replicates of the same condition by at least one of the five tests. It is defined as the proportion of *p*‐values less than 0.05 out of all runs. The summary of conditions where cluster structure was detected by this criterion is in Table 1. The (1)–(3) denote the replicates of a given condition.

**TABLE 1:** Conditions where clustering of survival cells was identified by statistical tests

	Hopkins's test	Classic dip	Classic Silverman	PCA dip	Dip‐dist	
	1000 runs	1000 runs	100 runs	1000 runs	1000 runs	
Images	*p* < 0.05	*p* < 0.05	*p* < 0.05	*p* < 0.05	*p* < 0.05	Number of particles
SL1344 20 min H_2_O_2_ 48 h (1)			100%			1521
SL1344 20 min H_2_O_2_ 48 h (2)			100%			1425
SL1344 20 min H_2_O_2_ 48 h (3)			100%			1515
SL1344 20 min NaClO 24 h (2)	100%	100%				98
SL1344 20 min NaClO 24 h (3)		100%			100%	162
SL1344 20 min NaClO 48 h (1)			99%			650
SL1344 20 min NaClO 48 h (2)			83%			591
SL1344 40 min NaClO 48 h (1)	100%	100%	100%		81.2%	751
SL1344 40 min NaClO 48 h (2)			100%			832
SL1344 40 min NaClO 48 h (3)	100%	100%	100%			685
SL1344 40 min PAA 24 h (1)		100%				28
SL1344 40 min PAA 24 h (2)				100%	100%	35
SL1344 40 min PAA 24 h (3)				100%		35
SL1344 40 min H_2_O_2_ 48 h (1)	97.8%	100%	95%			96
SL1344 40 min H_2_O_2_ 48 h (2)			100%			421
SL1344 40 min H_2_O_2_ 48 h (3)			100%			401
SL1344 48 h control (1)			99%			1307
SL1344 48 h control (2)			100%			1195
SL1344 60 min NaClO 48 h (1)	95.6%	100%	100%	100%		582
SL1344 60 min NaClO 48 h (3)			100%			382
3750 20 min PAA 24 h (1)			100%			47
3750 20 min PAA 24 h (3)		100%		100%		25

*Note*: The percentage represents the clusterability fraction, that is, the proportion of *p*‐values less than 0.05 out of all runs.

Altogether 8 out of the 19 conditions possessed a sufficient cluster structure and were meaningfully partitioned in at least one of the time points (24 h or 48 h). In Table 1, replicates without clustering structure were omitted, thus giving a total of 22 images across 8 conditions. All five tests had a very high clusterability fraction (>95%) except Classic Silverman and Dip‐dist for the SL1344 20 min NaClO 48 h (2) and SL1344 40 min NaClO 48 h (1) samples.

The Classic Dip and Silverman tests showed differences in clustering between conditions, for example, SL1344 20 min NaClO 24 h (2–3) samples have a relatively small number of sparsely distributed particles, and thus, methods that account for outlier robustness like Dip test, may be more effective. In contrast, SL1344 20 min H_2_O_2_ 48 h (1–3), SL1344 20 min NaClO 48 h (1–2) and SL1344 48 h control (1 and 3) samples had many uniformly distributed particles and as such, methods that allow for small clusters like Silverman test may be more appropriate (Adolfsson et al., 2017).

The Hopkins statistic performed consistently with the Classic Dip test, except for the 3750 20 min PAA 24 h condition. The PCA Dip and Dip‐dist methods had a high clusterability fraction (>80%) for at least one sample across four conditions (SL1344 20 min NaClO 24 and 48 h counted once). Although PCA Dip and Dip‐dist are mostly recommended for clustering of high‐dimensional data sets (Adolfsson et al., 2017), they were able to detect a clustering structure in samples where the Classic Dip was not. We conclude that classic multimodality tests complemented with their counterparts on reduced versions of the data, might be preferred to analyse the spatial distribution of live cells.

**FIGURE 4:**
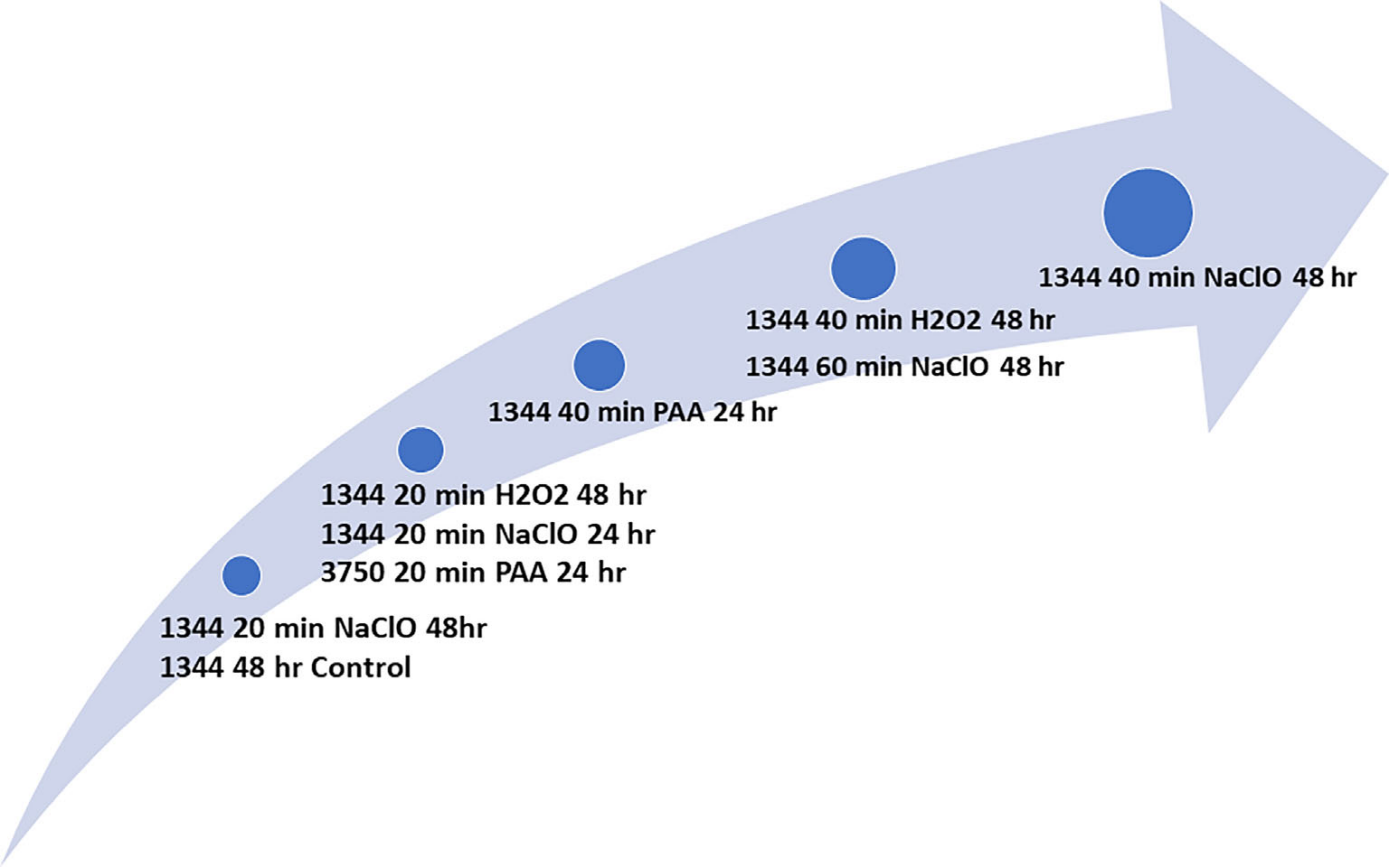
The relative degree of clustering observed in the eight conditions. This shows clustering was most likely in biofilms formed by SL1344 (weak) compared to 3750 (strong)

We assessed the degree of clusterability in each condition by the number of times high clusterability fraction (>80%) was detected across replicates by the 5 statistical tests in Table 1 (Figure 4).

### Clustering of survivors under stress is not random

Previous studies have shown a high level of bacterial survival in biofilms treated with NaClO and H_2_O_2_ (Flach et al., 2016; Lin et al., 2011). This was also the case here (Figure S4) and we identified a non‐uniform distribution of live cells by analysing confocal microscopy images. Figure 4 shows that SL1344 biofilms treated with biocides of medium to low potency (NaClO, H_2_O_2_) for 20–40 min possess an inherently clustered structure. Changing the length of dosing from 20 to 40 min affected clustering significantly for NaClO‐treated strain SL1344 biofilms at 48 h. The mean number of particles in an image was smaller for the 20 min than for the 40 min NaClO treatment (675.3 and 756, respectively). This is not surprising, given the reduced duration of exposure time to NaClO. The statistical tests also revealed that there is clustering of live cells present in untreated SL1344 biofilms at 48 h which becomes more marked. This phenomenon has been observed before in other single‐species biofilms (Kara et al., 2007) and is likely to reflect the underlying structure resulting from cells embedded in an extracellular polysaccharide matrix (EPS), which can stimulate cluster formation.

**FIGURE 5:**
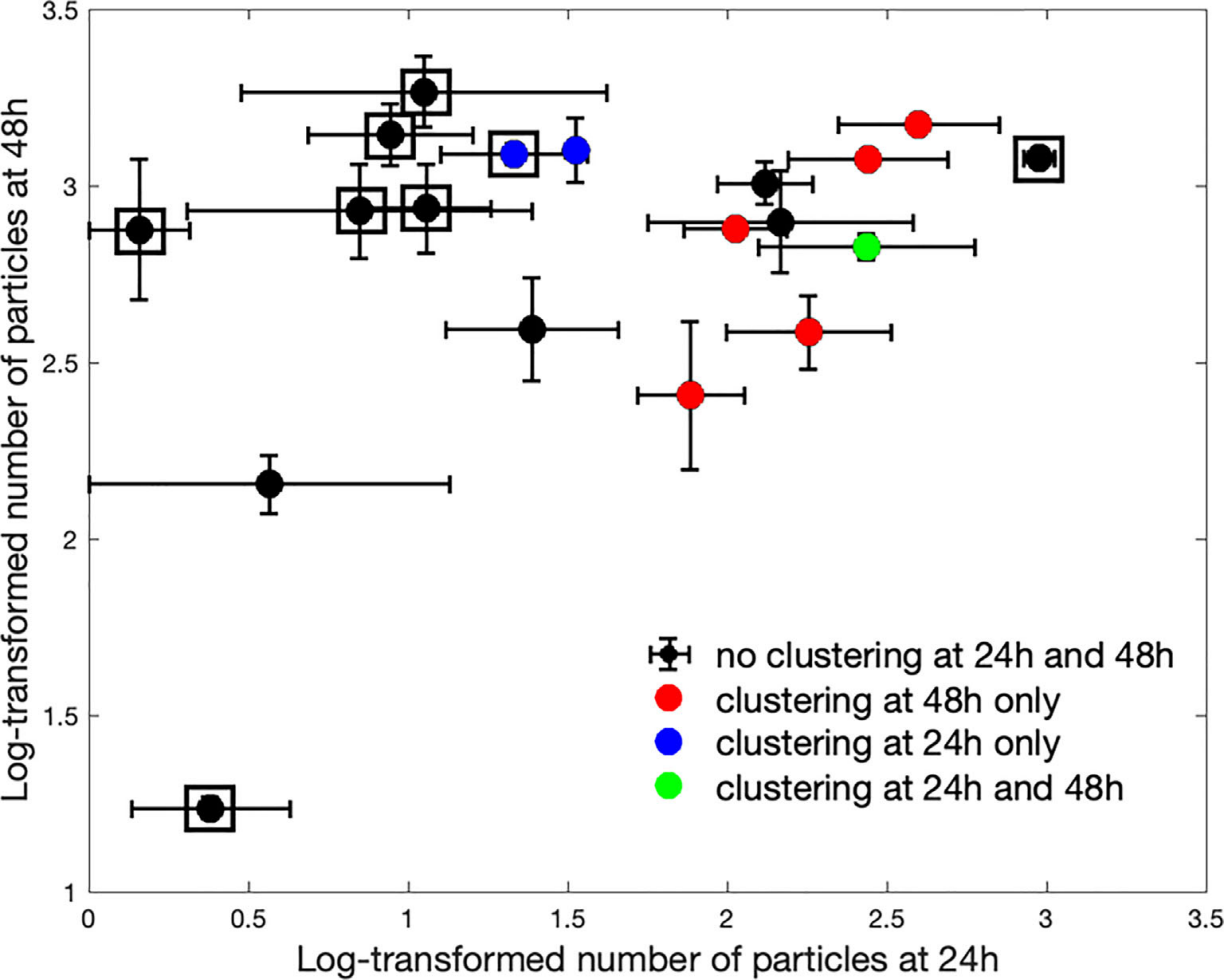
Mean and SE of the number of particles for 19 different conditions. Colours denote cell clusters at 24 h or 48 h. cluster structure is present in 72.7% of the conditions associated with strain SL1344 in at least one of the time points. Squares around the data points represent 3750 biofilms and points with no squares represent SL1344 biofilms

There was however a noticeable lack of clustering detected in 3750 biofilms except the 3750 20 min PAA 24 h condition, (which had a relatively low bacterial survival due to the high potency of PAA). In our system, time had a large impact on the clusterability of image data sets from 3750 biofilms exposed to biocides of medium to low potency. This may reflect the decrease in biocide efficacy against the more robust biofilms formed by 3750 for 48 h compared to 24 h, resulting in a low level of selection for survivors which would be consistent with some other studies (Stewart, 2015). We postulate that the formation of cell clusters provides protection against less potent oxidative biocides and that this becomes more pronounced as exposure is prolonged. Clustering was also more obvious in weaker biofilm forming strains where fewer cells are surviving. The transient (24 h) and more mature (48 h) cell clusters are depicted in Figure 5 (using the same data as depicted in Figure 2).

Given the identical treatment conditions between the two strains, one unexpected result in Figure 5 is that at 24 h strain SL1344 biofilms appear to survive biocide exposure better than strain 3750. This is an anomalous result since the opposite would be expected given the relatively weaker biofilm formation of this strain (Figure 2 and S1). This transient growth advantage might be linked to metabolic switching observed in Martins et al. (2013) and consequently, increased growth and cell aggregation to withstand concentrations of oxidizing biocides.

**FIGURE 6:**
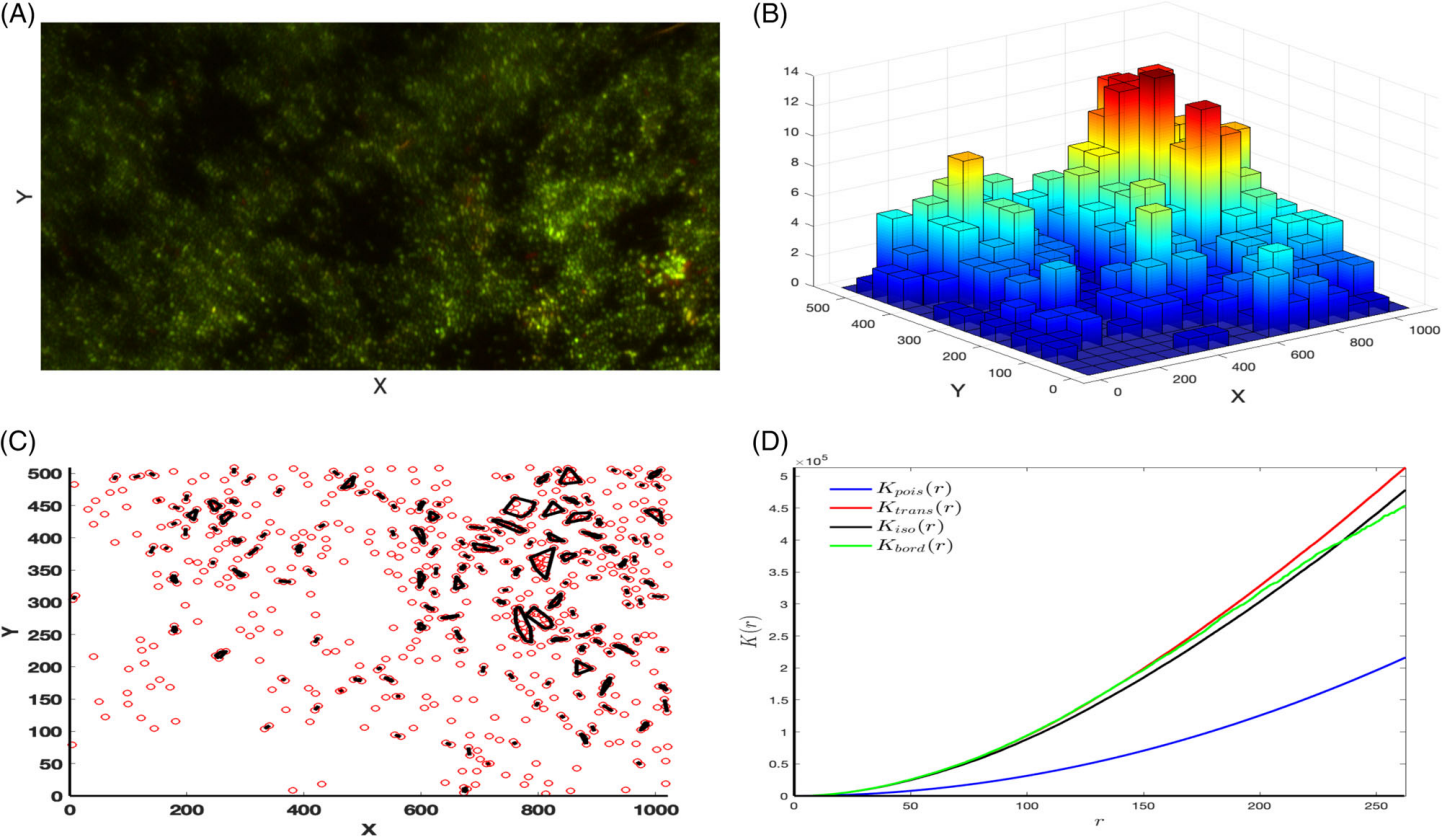
(A) Confocal image (maximum projection Z‐stack) of replicate (3) from the most clusterable condition 1344 40 min NaClO 48 h. (B) 3D histogram representing the number of green channel particles extracted from z‐slices from the same condition with a maximum of 13 particles in a bin. (C) Plots of the clusters enclosed by their convex hulls, at the intrinsic distance dI = 12.8218, following the methodology developed in Unis (2010). (D) Ripley's *K*‐function Kpois compared with estimates of the *K*‐function based on different edge correction methods: Translation correction (Ktrans), Ripley's isotropic correction (Kiso), border correction (Kbord), using the Kest function from the spatstat R package. Deviations between the empirical *K* curves and the true value of *K* for a completely random (Poisson) point process, Kpois = *πr* ^2^, may suggest spatial clustering

Analysis of single images from a biofilm can be complicated by blurring as they are taken as slices through a three‐dimensional structure, 3D or 2D visualization of clusters can help resolve this and help analysis of whether clusters are spaced randomly. Figure 6A shows the green channel (live cells) of SL1344 grown for 48 h and exposed to NaClO for 40 min. Figure 6B shows the 3D histogram of the *Z*‐stack image, the number of bins was chosen such that the maximum number of particles in a bin is 13, as suggested by a novel methodology based on hierarchical clustering (Espinoza et al., 2012) that quantifies the numbers and sizes of clusters by computing an intrinsic distance. Two points belong to the same cluster if they are closer than this distance. This method was used in Figure 6C, where the clusters are enclosed by their convex hulls. Clustering analysis indicates that 456 out of the total 685 data points are in clusters, at the intrinsic distance *d*_*I*_ = 12.8218, and the three largest cluster sizes are 13, 11 and 10. About 67.5% of the 456 extracted data points were in clusters of sizes 2–4, and there were 21 clusters of sizes at least 5. This is in line with observations that mono‐species biofilms of *Salmonella* tend to form scattered single‐cell or small clusters (Gonzalez‐Machado et al., 2018; Pang et al., 2017).

Spatial point pattern analysis allows us to classify spatial distributions of the green channel particles in Figure 6A. A powerful tool for examining spatial independence across scales is Ripley's *K*‐function which has been applied to quantify the spatial distribution of bacteria within the biofilm in several studies (Hart et al., 2019; Ishkov et al., 2021; Marchal et al., 2017). The univariate form of Ripley's K‐function (where only one type of point is considered) is.

$$\bar{K}\left(r\right)=\frac{A}{n^{2}}\sum_{i\neq j}^{n}w_{\mathrm{ij}}\left(r\right)I_{\mathrm{ij}}\left(r\right),$$

where *n* is the number of points inside a region of area *A*, $w_{\mathrm{ij}}$ is the edge effect correction factor. The indicator function $I_{\mathrm{ij}}$ defines whether a point $p_{j}$ is inside a neighbourhood *r* of point $p_{i}$ or not based on the Euclidean distance between the points $p_{i}$ and $p_{j}$. Appropriate edge correction can improve the power of the statistical tests (Yamada & Rogerson, 2003). We used the spatstat R package (https://cran.r-project.org/web/packages/spatstat/index.html) which has different estimates of Ripley's *K*‐function built in. Since these curves lie above the theoretical *K*‐function $K\left(r\right)=\pi r^{2}$, the point pattern in Figure 6D is clustered.

### Live and dead cells are not colocalized

We used Imaris 14.0.0 (Bitplane, South Windsor, CT, USA) to quantify colocalization between live and dead cells on a given Z‐stackimage in order to understand whether distinct clusters differed in their chances of survival. The colocalization uses a statistical approach developed by Costes et al. (2004), which is done by estimating simultaneously the maximum threshold of intensity for the green and red channels below which particles exhibit no correlation. The main advantage of Costes method is that it automatically quantifies colocalization in any region of the image without user intervention. Imaris has been widely used to analyse image stacks for Salmonella in a variety of biofilm and immune host (Burton et al., 2014) settings. We used two main methods of colocalization analysis for the significant conditions, co‐occurrence and correlation. Co‐occurrence‐based colocalization analyses such as Mander's coefficient, determine the extent of spatial overlap between green and red fluorescent channels. Correlation‐based colocalization analyses such as Pearson's coefficient, describe the degree to which the abundance of the spatially overlapping channels are related to each other. Both approaches have different strengths and weaknesses (Aaron et al., 2018).

Table S2 summarizes the Imaris output features for the clusterable confocal images such as quantification of colocalization between the two channels and for the individual channels green and red channel thresholds. There was no evidence of colocalization between the two channels, suggesting that live and dead bacteria establish spatially distinct regions within a *Salmonella* biofilm. This shows that clustering of survivors is not simply an artefact of where cells have produced most biomass. The colocalization of green channel particles alone was above 46% for all clusterable confocal images, except SL1344 20 min NaClO 24 h (2–3) and 3750 20 min PAA 24 h (1 and 3). The colocalization of the corresponding red channel particles was under 35%, except for SL1344 20 min NaClO 48 h (1) and SL1344 40 min PAA 24 h (2).

The Pearson correlation coefficient (PCC) expresses to what degree signal intensity variation in green channel can be explained by the related variation in the red channel, assuming a linear relationship. Consistently medium‐to‐high positive PCC values were measured for SL1344 20 min H_2_O_2_ 48 h (1–3), SL1344 20 min NaClO 48 h (1–2), SL1344 40 min NaClO 48 h (1–3), SL1344 40 min H_2_O_2_48 h (1–3) cases (PCC >0.4). Interestingly, all these cases occur in SL1344 biofilms treated at 48 h with biocides of medium to low potency for 20–40 min.

The Mander's coefficient accounts for the signal intensity of particles in each of the channels. M_g_ is the co‐occurrence fraction of green colour with red colour and vice versa for M_r_. Except for SL1344 20 min NaClO 24 h (3) and SL1344 40 min PAA 24 h (2), all other clusterable confocal images have M_g_ <0.24 and M_r_ <0.19. These results indicate a low extent of co‐occurrence between live cells (and similarly, for dead cells) showing initial seeding is probably not related to final viability.

## CONCLUSIONS

This work demonstrates in controlled conditions the differences in efficacy between three common oxidizing biocides against biofilms of 2 *Salmonella* strains and established that bacterial survival is strain, exposure time and biocide dependent. This shows that designing effective biocidal regimes requires data from diverse strains to ensure adequate coverage of potentially more tolerant strains when in a biofilm context. Improving our understanding of differences in how strains respond to biocidal challenges shows that testing biocide regimes should include use of strains with different biofilm formation capacities as well as different biocide concentrations to predict efficacy.

One of the most popular supervised learning methods, SVM with RBF kernel, has been applied for binary classification of strains as weak or strong biofilm formers. We conducted 10‐fold and leave‐out‐one cross‐validations to test the model, and performed SVM assessment, by evaluating sensitivity and specificity. We note that increasing sensitivity and specificity lowers the probability of type II and type I error, respectively. We expect other algorithms to perform similarly and although we did not show the results, we also tried a random forest which resulted in a similar performance. (With only 18 data points, we do not anticipate classification algorithms to vary a lot in performance.) Given 3D confocal microscopy data of thick biofilms and enough samples for given biofilm‐forming strains or treatments, a PCA‐SVM classifier might also be used to extend the analysis. A similar technique involving different antibiotics has been applied in Yoram et al. (2018).

Most statistical approaches addressing the spatial distribution of biofilm cells rely on spatial analysis methods such as Ripley's *K*, and to a lesser extent on methods of spatial randomness such as Hopkins statistic. In addition to these two methods, we applied multimodality tests (Classic Dip test and Classic Silverman test) and related tests on reduced versions of the data (PCA Dip and Dip‐dist), supported by clusterability fraction, to evaluate clusterability in both strains. Although computationally expensive, the advantage of our approach is to calculate the *clusterability fraction*, that could be compared across different conditions, at the same level of significance. We hope to extend this approach to coupling data describing particle counts and certain chemical entities (e.g., metabolites, autoinducers), obtained by confocal microscopy.

Survival within biocide treated biofilms was not uniform which supports previous work suggesting large numbers of cells are sacrificed in a process of impeding biocide penetration (Diez‐Garcia, 2012). However, clustering of survivors was seen even in relatively immature biofilms (e.g., SL1344 at 24 h) which makes differential susceptibility between clusters more likely than a physical protective effect. The clustering observed is likely to reflect differences between clusters that are imprinted as the initial seeding of microcolonies by individual cells. Differences in genotype, gene expression or other epigenetic features which vary between clusters are probably related to likelihood of survival.

This study demonstrates clear differential behaviour of clusters of cells in terms of survival within biofilms as well as outlining a framework to identify and quantify clusters of live or dead cells. The mechanistic basis which dictates differential survival of these clusters is uncertain and future work to study gene content, expression and behaviour of single cells within clusters is needed. Understanding how clusters establish, differentiate from each other and survive stress will help develop strategies to control biofilm formation and is an important future goal.

## AUTHOR CONTRIBUTIONS

KG performed experiments and data analysis and wrote the paper, TW performed analysis of data, AA performed statistical analysis, JY‐M designed the study and wrote the paper, BS designed the study, performed analysis and wrote the paper, MW designed the study and wrote the paper.

## CONFLICT OF INTEREST

The authors declare no competing financial or non‐financial interests.

## Supporting information


**Appendix S1:** Supporting information

## Data Availability

All images used in this study are stored on servers at Quadram Institute Bioscience and are freely available upon request to the corresponding author.
