# Supplementary material for: Responses of Salmonella biofilms to oxidizing biocides: Evidence of spatial clustering
Source: Environ Microbiol. 2022 Nov 6;24(12):6426–38. doi: 10.1111/1462-2920.16263 (PMC10099496; doi:10.1111/1462-2920.16263)
Supplement: Supplementary file 1 — Appendix S1: Supporting information [file EMI-24-6426-s001.docx]

**Supplementary information**

***Supplementary tables***

**Table S1.** Images used for clustering analysis

| **Images at 24h** | **Images at 48h** |
| --- | --- |
| 1344 20 min H2O2 24 hr | 1344 20 min H2O2 48 hr |
| 1344 20 min NaClO 24 hr | 1344 20 min NaClO 48 hr |
| 1344 20 min PAA 24 hr | 1344 20 min PAA 48 hr |
| 1344 40 min H2O2 24 hr | 1344 40 min H2O2 48 hr |
| 1344 40 min NaClO 24 hr | 1344 40 min NaClO 48 hr |
| 1344 40 min PAA 24 hr | 1344 40 min PAA 48 hr |
| 1344 60 min H2O2 24 hr | 1344 60 min H2O2 48 hr |
| 1344 60 min NaClO 24 hr | 1344 60 min NaClO 48 hr |
| 1344 60 min PAA 24 hr | 1344 60 min PAA 48 hr |
| 1344 24 hr Control | 1344 48 hr Control |
| 1344 24 hr EtOH | 1344 48 hr EtOH |
| 3750 20 min NaClO 24 hr | 3750 20 min NaClO 48 hr |
| 3750 20 min PAA 24 hr | 3750 20 min PAA 48 hr |
| 3750 40 min NaClO 24 hr | 3750 40 min NaClO 48 hr |
| 3750 40 min PAA 24 hr | 3750 40 min PAA 48 hr |
| 3750 60 min NaClO 24 hr | 3750 60min NaClO 48hr |
| 3750 60 min PAA 24 hr | 3750 60 min PAA 48 hr |
| 3750 24 hr Control | 3750 48 hr Control |
| 3750 24 hr EtOH | 3750 48 hr EtOH |

**Table S2. Summary of features of clustering from Imaris outputs.**

| **Images** | **% of all particles above threshold**  **colocalized** | **% of green**  **channel**  **particles above**  **threshold**  **colocalized** | **% of red**  **channel**  **particles**  **above**  **threshold**  **colocalized** | **Pearson’s**  **coefficient**  **for area above**  **threshold**  **colocalized** | **Thresholded**  **Mander’s coefficient**  **for green**  **channel (M_g_)** | **Thresholded**  **Mander’s coefficient**  **for red**  **channel (M_r_)** |
| --- | --- | --- | --- | --- | --- | --- |
| 1344 20 min H2O2 48 hr (1)  1344 20 min H2O2 48 hr (2)  1344 20 min H2O2 48 hr (3) | 0.23  0.56  0.05 | 90.87  94.12  83.97 | 18.04  34.11  23.22 | 0.5426  0.7936  0.8659 | 0.1229  0.046  0.0078 | 0.0123  0.02  0.0026 |
| 1344 20 min NaClO 24 hr (2)  1344 20 min NaClO 24 hr (3) | 0  4.82 | 8.7  16.52 | 0.22  66.77 | -1  0.4717 | 0.0141  0.272 | 0.0002  0.5551 |
| 1344 20 min NaClO 48 hr (1)  1344 20 min NaClO 48 hr (2) | 0.11  0.26 | 46.92  93.98 | 53.32  23.09 | 0.4711  0.6191 | 0.0069  0.0355 | 0.0105  0.0114 |
| 1344 40 min NaClO 48 hr (1)  1344 40 min NaClO 48 hr (2)  1344 40 min NaClO 48 hr (3) | 0.19  0.32  0.24 | 84.26  88.07  92.64 | 27.51  21.83  28.55 | 0.6952  0.6012  0.6261 | 0.0289  0.0439  0.0344 | 0.0112  0.0137  0.0139 |
| 1344 40 min PAA 24hr (1)  1344 40 min PAA 24hr (2)  1344 40 min PAA 24hr (3) | 0.13  2.87  0.02 | 48.17  53.12  85.42 | 22.86  72.61  4.68 | 0.0477  0.5414  0.3017 | 0.0827  0.8365  0.0744 | 0.0669  0.9098  0.0093 |
| 1344 40 min H2O2 48 hr (1)  1344 40 min H2O2 48 hr (2)  1344 40 min H2O2 48 hr (3) | 0.01  0.01  0.02 | 77.78  80.95  73.11 | 3.59  12.5  16.86 | 0.766  0.5318  0.4023 | 0.02  0.008  0.0087 | 0.003  0.0017  0.0027 |
| 1344 48 hr Control (1)  1344 48 hr Control (3) | 0  0.01 | 100  100 | 0.03  0.07 | 0  0 | 0.2069  0.2388 | 0.0001  0.0003 |
| 1344 60 min NaClO 48hr (1)  1344 60 min NaClO 48hr (3) | 0.01  0 | 84.44  100 | 1.94  3.05 | -0.128  0.6447 | 0.0216  0.0069 | 0.001  0.0005 |
| 3750 20 min PAA 24 hr (1)  3750 20 min PAA 24 hr (3) | 0.06  0 | 0.72  0 | 57.84  0 | -0.0358  0 | 0.003  0 | 0.1816  0.19 |

**Supplementary figures**

**Figure S1. Different biofilm formation capacity of the two strains. Panel A** shows a comparison of biofilm formation by both strains over time, panel **B** shows results from crystal violet staining of biofilms formed in microtitre trays (n=24 per strain) and panel **C** shows analysis of percentage coverage of the surface of the microscope viewing chamber on Bioflux plate (n=12). Grown in MOPS based minimal media at 20^o^C for either 24, 48, 72 or 96 hrs. The error bars denote the standard deviation from the mean.

**
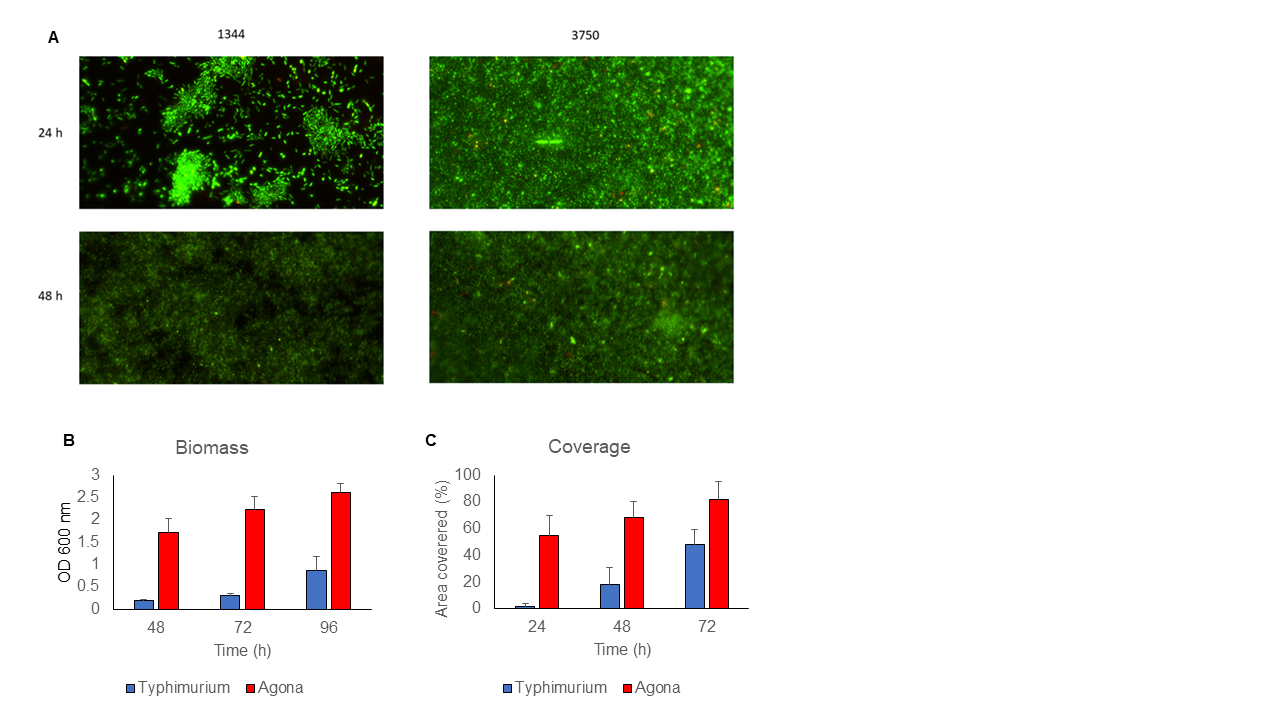
**

**Figure S2**. ROC curve for training and test data provides accurate results for SVM with γ=0.27, *C*=12. The training and test results show that there is no over-fitting. γ=0.27, *C*=12 in **A** and γ=0.8, *C*=5 in **B**.

**A**


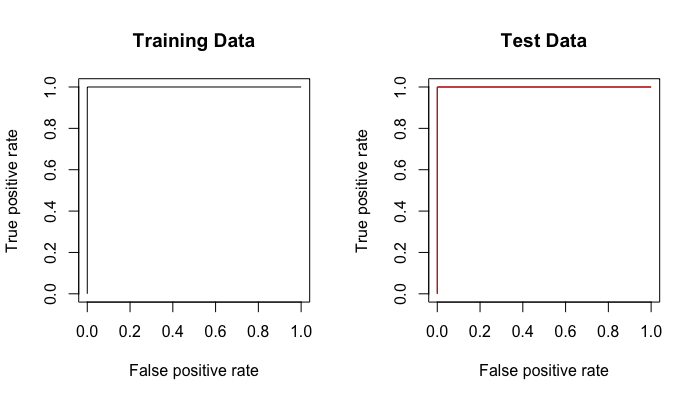


**B**

**
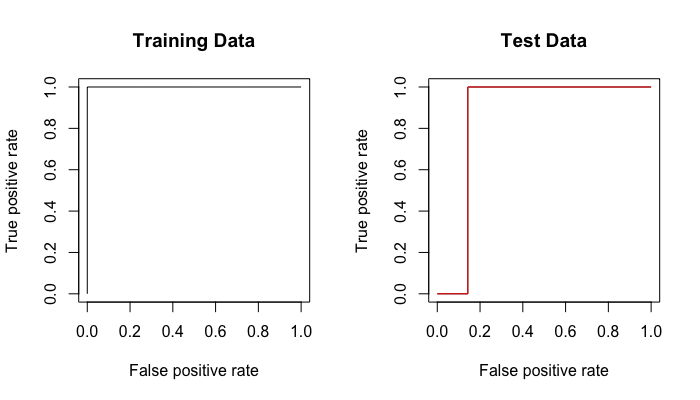
**

**Figure S3. Response to different biocides** Confocal microscopy images of *Salmonella enterica* serovar Agona exposed to hydrogen peroxide, peracetic acid and sodium hypochlorite for 20, 40 or 60 mins. *Salmonella* biofilms were grown in a microfluidic flow system for either 24 (panel **A**), or 48 hrs (panel **B**) at 20^o^C. Cells were stained with PI and STYO9; live cells are green and dead cells red.


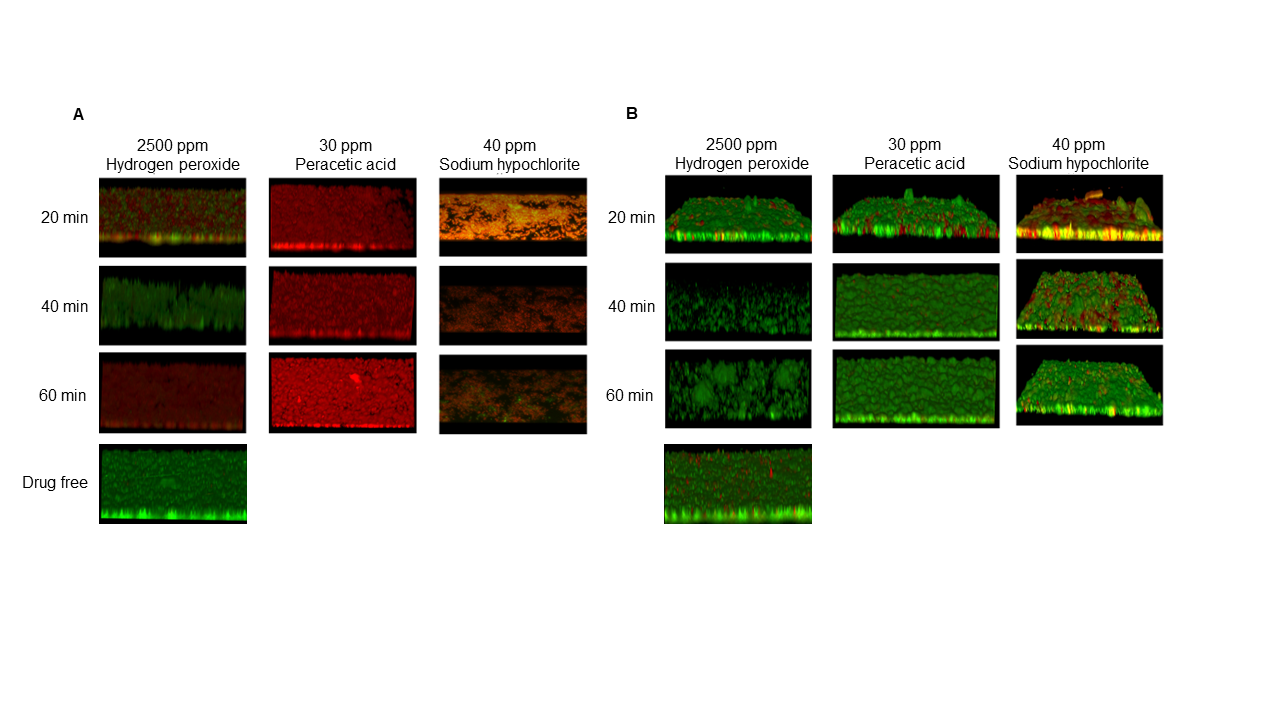


**Figure S4. Viability after exposure to different biocides:** Viable numbers of *Salmonella enterica* serovar Agona recovered from biofilms after being exposed to hydrogen peroxide (**A**), peracetic acid (B) and sodium hypochlorite (**C**) for 20, 40 or 60 mins. Points indicate the average of 5 replicate values and error bars show standard deviation.

**
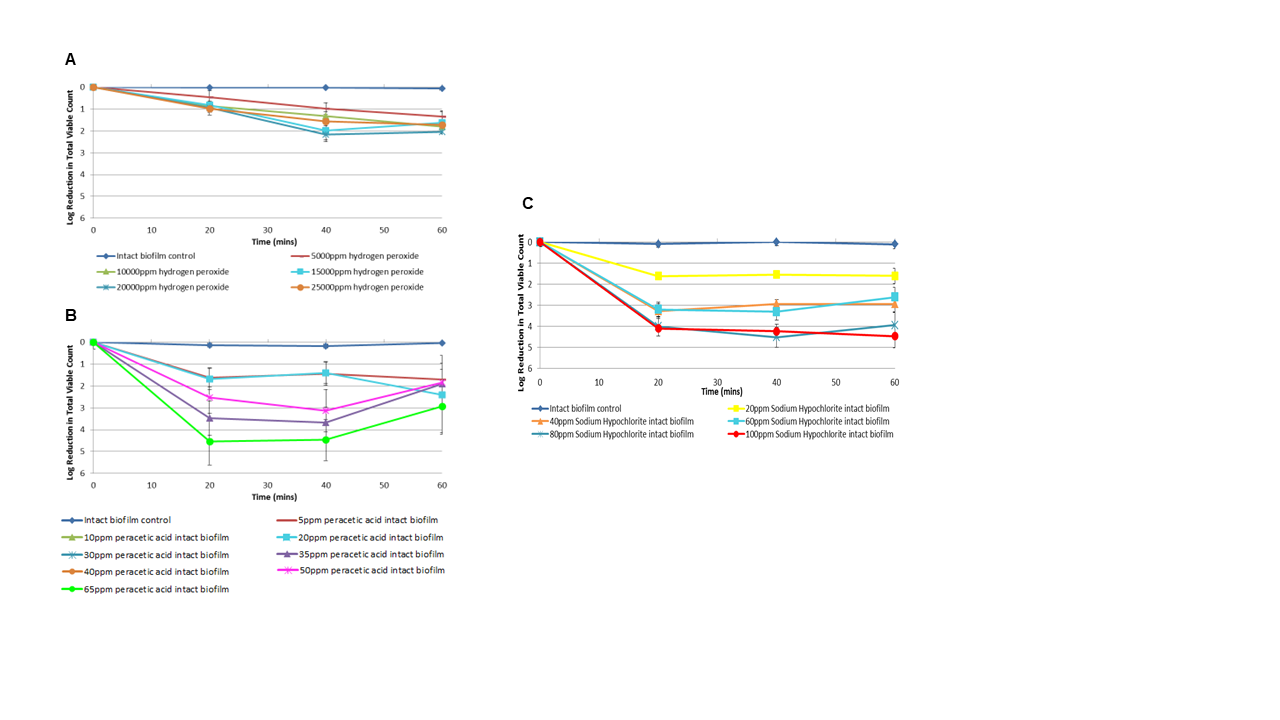
**
